# Renal Involvement in Down Syndrome: Developmental and Clinical Perspectives

**DOI:** 10.3390/diagnostics16213630

**Published:** 2026-11-03

**Authors:** Bojana Andrejić Višnjić, Maja Samardžić Lukić, Vesna Stojanović, Sandra Trivunić Dajko, Milena Šunjević, Nataša Kovač, Milana Bosanac

**Affiliations:** 1 Faculty of Medicine, University of Novi Sad, 21000 Novi Sad, Serbia; bojana.andrejic-visnjic@mf.uns.ac.rs (B.A.V.); maja.samardzic-lukic@mf.uns.ac.rs (M.S.L.); vesna.stojanovic@mf.uns.ac.rs (V.S.); sandra.trivunic-dajko@mf.uns.ac.rs (S.T.D.); milena.sunjevic@mf.uns.ac.rs (M.Š.); natasa.kovac@mf.uns.ac.rs (N.K.); 2 Institute for Child and Youth Health Care of Vojvodina, 21000 Novi Sad, Serbia; 3 Center for Pathology and Histology, University Clinical Center of Vojvodina, 21000 Novi Sad, Serbia

**Keywords:** Down syndrome, CAKUT, nephrogenesis, chronic kidney disease, glomerular diseases, renal biomarkers

## Abstract

With the increasing life expectancy of individuals with Down syndrome (DS), kidney disease has emerged as an important yet under-recognized contributor to long-term morbidity and mortality in this population. This narrative review summarizes current knowledge regarding the developmental, structural, histopathological, and clinical aspects of renal involvement in DS. A literature search was conducted, and studies addressing renal structure, function, development, or pathology in individuals with DS (clinical studies, autopsy studies, case reports, and experimental studies) were included. Available evidence indicates that renal involvement in DS extends beyond congenital anomalies to include altered nephrogenesis, acquired glomerulopathies, and progressive functional impairment. Histopathological studies of fetal kidneys suggest altered nephrogenesis and delayed renal maturation in DS, potentially resulting in reduced nephron endowment. However, the long-term functional consequences of these developmental findings remain uncertain. Reported acquired glomerular diseases are heterogeneous and include IgA nephropathy, focal segmental glomerulosclerosis, membranous nephropathy, ANCA-associated glomerulonephritis, and several rarer entities. Conventional renal biomarkers may underestimate kidney dysfunction in DS because of altered body composition and reduced muscle mass, while evidence regarding novel biomarkers such as NGAL and KIM-1 remains limited. Renal involvement in DS is multifactorial and likely reflects the interplay between disturbed nephrogenesis, developmental renal abnormalities, and acquired renal disease, resulting in increased renal vulnerability throughout life. Improved awareness, earlier nephrological surveillance, and validation of sensitive renal biomarkers may facilitate earlier detection of kidney injuries and improve long-term renal outcomes in individuals with DS.

## 1. Introduction—Discovery, Etiology, and Epidemiology of Down Syndrome

Down syndrome (DS), or trisomy 21, is a genetic disorder first described in the late nineteenth century by the English physician John Langdon Down [1]. Almost 100 years later, Raymond Turpin’s laboratory in Paris established the association between DS and chromosome 21 [2]. DS results from the presence of all or part of an extra copy of chromosome 21 and is the most common survivable autosomal aneuploidy in humans [3].

Down syndrome occurs in approximately one in 600–800 live births worldwide, without a significant predilection for ethnicity or socioeconomic status, although its incidence increases with advancing maternal age [4]. The prevalence of DS in Europe is approximately 10.1 per 10,000 live births and has been estimated to reach 21.7 per 10,000 live births in the absence of elective pregnancy termination [5]. Trisomy 21 is associated with an increased risk of spontaneous fetal loss, although postnatal survival is substantially higher than that associated with other autosomal trisomies [2,3].

In addition to characteristic phenotypic features and intellectual disability, individuals with DS commonly exhibit somatic growth retardation and multisystem involvement, including congenital anomalies, endocrine abnormalities, immune dysregulation, hematological disorders, and metabolic disturbances, all of which contribute to increased morbidity and healthcare burden throughout life [6,7].

Renal health has become increasingly relevant in individuals with DS as advances in medical care have extended life expectancy. At the same time, congenital anomalies of the kidney and urinary tract (CAKUT) may remain underrecognized, while assessment of renal function in children with DS presents additional challenges related to their specific clinical characteristics [5].

### Down Syndrome, Kidney and Kidney Diseases

Since Berg et al. first described the association between Down syndrome and kidney and urinary tract abnormalities in 1960 [8], researchers and clinicians have shown increasing interest in this field. Renal dysfunction in individuals with DS may contribute to the development of chronic kidney disease (CKD). The causes of renal impairment in children with DS may be broadly categorized as primary or secondary. Early detection and, where possible, prevention are important, as certain renal abnormalities may predispose affected individuals to CKD later in life [7].

Studies on renal and/or urinary tract anomalies in DS primarily rely on autopsy and population-based investigations, although evidence also comes from case reports, cross-sectional studies, and retrospective cohort studies. All of these study types have limitations related either to their methodology or to the period in which they were conducted. Older studies relied on phenotypic descriptions to identify children with DS, while karyotyping markedly improved diagnostic accuracy; however, access to cytogenetic testing has historically varied across regions and healthcare systems [5]. The limited diagnostic capabilities of early ultrasound technology, differences in verification methods, and restrictions on the number of anomalies reported per case also contributed to significant heterogeneity and underreporting of congenital anomalies of the kidney and urinary tract (CAKUT). Despite these constraints, each study and report has contributed significantly to our current understanding of renal involvement in DS.

Although renal abnormalities have been repeatedly described in individuals with DS, previous reviews have focused primarily on the prevalence and clinical spectrum of renal and urological abnormalities or the genetic basis of CAKUT [4,5,7]. The present review adopts an integrated developmental, histopathological, and clinical perspective, bringing together evidence on fetal nephrogenesis, congenital renal anomalies, acquired glomerular diseases, and renal function assessment across the lifespan. It distinguishes established morphological and clinical findings from preliminary associations and mechanistic hypotheses, especially regarding the potential relationship between developmental renal abnormalities and subsequent renal dysfunction. By critically evaluating the strength and limitations of the available evidence, this review aims to identify clinically relevant uncertainties and priorities for future research.

## 2. Materials and Methods

### 2.1. Search Strategy

A literature search was conducted using the National Library of Medicine (PubMed) and Web of Science databases supplemented by manual screening of reference lists from relevant publications. We initially collected the literature in April 2024 and updated it in October–November 2025.

To improve transparency and reproducibility of the search process and identify additional relevant publications, updated searches were performed in both databases in October 2026. The searches combined terms related to Down syndrome with terms related to renal development, congenital anomalies, and kidney diseases using Boolean operators (AND/OR). Medical Subject Headings (MeSH) and title/abstract fields were used in PubMed, whereas the Topic (TS) field was used in Web of Science Core Collection.

The following database-specific search strategies were applied:

(a) PubMed:

(“Down Syndrome”[MeSH Terms] OR “Down syndrome”[Title/Abstract] OR “Down’s syndrome”[Title/Abstract] OR “trisomy 21”[Title/Abstract] OR “trisomy G”[Title/Abstract]) AND (“Kidney”[MeSH Terms] OR “Kidney Diseases”[MeSH Terms] OR “Kidney Abnormalities”[MeSH Terms] OR kidney[Title/Abstract] OR kidneys[Title/Abstract] OR renal[Title/Abstract] OR nephro*[Title/Abstract] OR glomerul*[Title/Abstract] OR “urinary tract”[Title/Abstract] OR CAKUT[Title/Abstract] OR nephrogenesis[Title/Abstract] OR metanephros[Title/Abstract]).

(b) Web of Science Core Collection:

TS=(“Down syndrome” OR “Down’s syndrome” OR “trisomy 21” OR “trisomy G”) AND TS=(kidney OR kidneys OR renal OR nephro* OR glomerul* OR “urinary tract” OR CAKUT OR nephrogenesis OR metanephros).

The updated searches identified 764 records in PubMed and 574 records in Web of Science Core Collection, yielding a total of 1338 records before deduplication and screening. No publication date, language, or document-type filters were applied. After removing 411 duplicates, 927 unique records remained for relevance assessment.

### 2.2. Inclusion and Exclusion Criteria

We screened records identified through database searches for relevance based on titles and abstracts, then examined full texts of potentially relevant publications when available. Reference lists of relevant publications were additionally examined to identify studies not retrieved through database searches.

Studies reporting data on renal development, morphology, congenital anomalies, renal function, or acquired kidney diseases in individuals with Down syndrome were considered eligible. Clinical and observational studies, autopsy and histopathological studies, case reports, and case series were considered when they provided relevant information on renal involvement in this population. Relevant experimental studies and publications addressing normal nephrogenesis or molecular mechanisms of renal development were also considered to provide the necessary embryological and pathophysiological context.

Publications whose full text was not available were excluded from further consideration, as were congress abstracts. Studies focused exclusively on disorders of the lower parts of the urinary tract or reproductive organ disorders without reported renal involvement, papers that dealt with dialysis or other therapeutic procedures without relevant data on renal pathology or function, as well as case reports focused primarily on treatment, without corresponding data on renal damage, were excluded. Additional reasons for exclusion were studies of renal biomarkers in pregnant women without assessment of fetal renal development or function, studies of prenatal screening for chromosomal anomalies that did not include assessment of fetal kidneys, as well as publications in which Down’s syndrome or renal diseases were only mentioned in passing, without data relevant to the topic of this review. The final selection of the literature was based on the publications’ relevance to the narrative review.

No strict language restrictions were applied. Publications in languages other than English were considered when relevant information was available in English-language abstracts or other reliable sources. For selected historical publications whose original full texts could not be accessed, we used information reported in relevant reviews and explicitly acknowledged the secondary source.

The relevance of the identified publications was assessed by two authors, and any disagreements were resolved by joint discussion, with the consultation of co-authors with the greatest expertise in the field to which the publication in question referred.

Of the 98 references cited in the final manuscript, 48 were identified through the database searches, while the remaining references comprised 41 additional publications identified through reference list screening, five historical studies reported through secondary sources, and four books or book chapters.

### 2.3. Data Extraction and Synthesis

Study selection was primarily conducted by one author, while publications addressing renal function assessment and emerging biomarkers were selected by another co-author, according to their respective areas of expertise.

Data were extracted manually and organized into thematic domains, including congenital renal anomalies, nephrogenesis and developmental findings, renal function assessment, histopathological abnormalities, and chronic kidney disease. Information regarding study design, patient population, diagnostic methods, and principal renal findings was synthesized narratively.

Because of substantial heterogeneity among study designs, populations, and outcome measures, quantitative synthesis and meta-analysis were not performed. Given the narrative nature of this review, no formal risk-of-bias assessment was conducted.

The review was designed as a comprehensive organ-focused synthesis integrating developmental, structural, functional, and pathological aspects of renal involvement in Down syndrome.

### 2.4. Preparation of Illustrations

**Figure 1:**
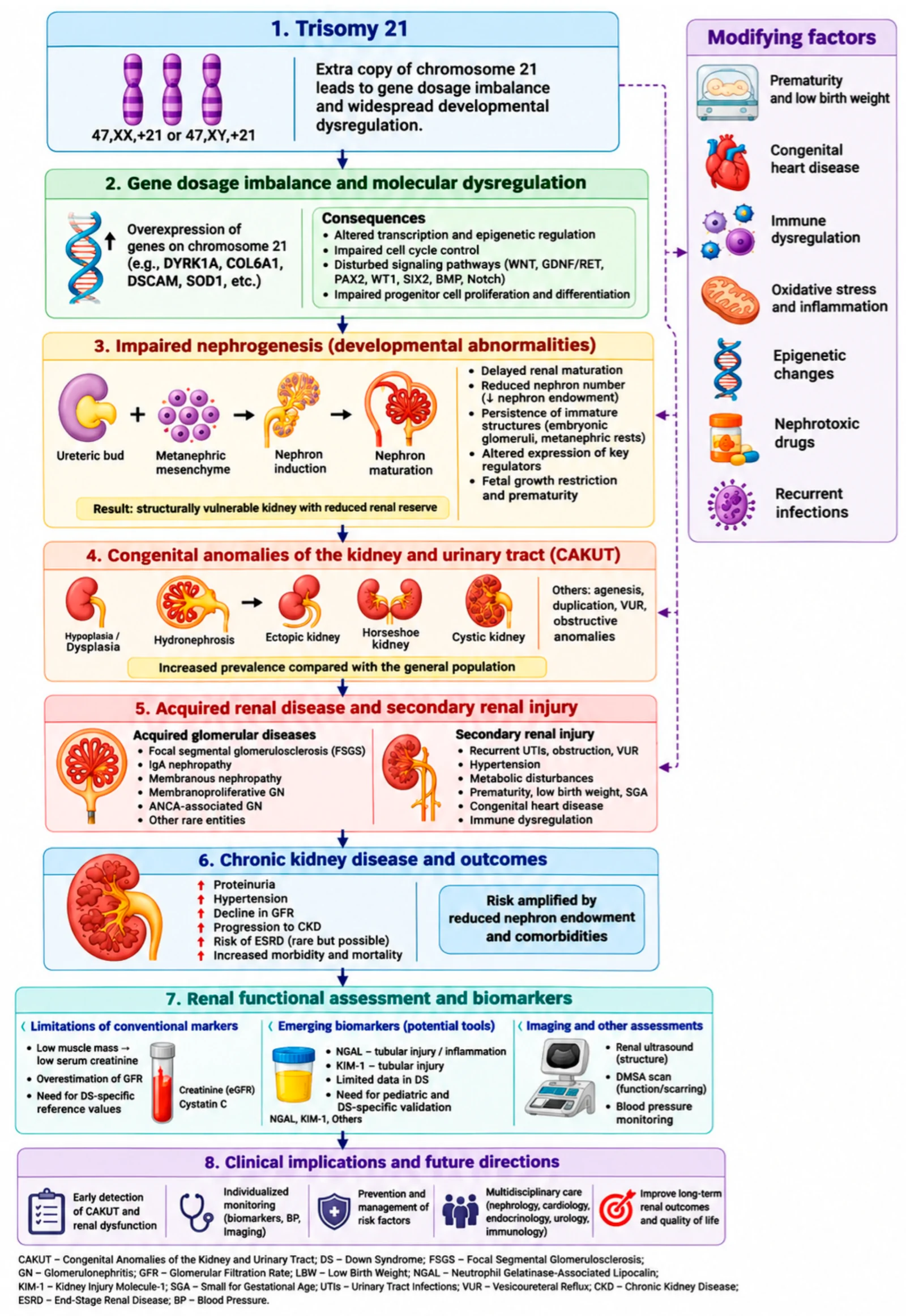
Proposed conceptual model of renal involvement in Down syndrome: from trisomy 21 to chronic kidney disease. The figure summarizes the proposed sequence of developmental and pathological events discussed in this review and potential implications for renal monitoring and future research. Solid arrows indicate the proposed sequence of developmental and clinical events, whereas dashed arrows represent the potential influence of modifying factors on renal abnormalities and disease progression. (The conceptual framework was developed by the authors based on the literature reviewed, and the illustration was generated with the assistance of ChatGPT (GPT-6, OpenAI, San Francisco, CA, USA; https://chatgpt.com). The authors reviewed and verified the scientific content of the illustration.).

Figure 1 was developed as an original conceptual illustration with the assistance of ChatGPT (GPT-6, OpenAI, San Francisco, CA, USA; https://chatgpt.com), based on the scientific evidence discussed in this review. The authors reviewed the generated illustration for scientific accuracy and consistency with the manuscript.

**Figure 2:**
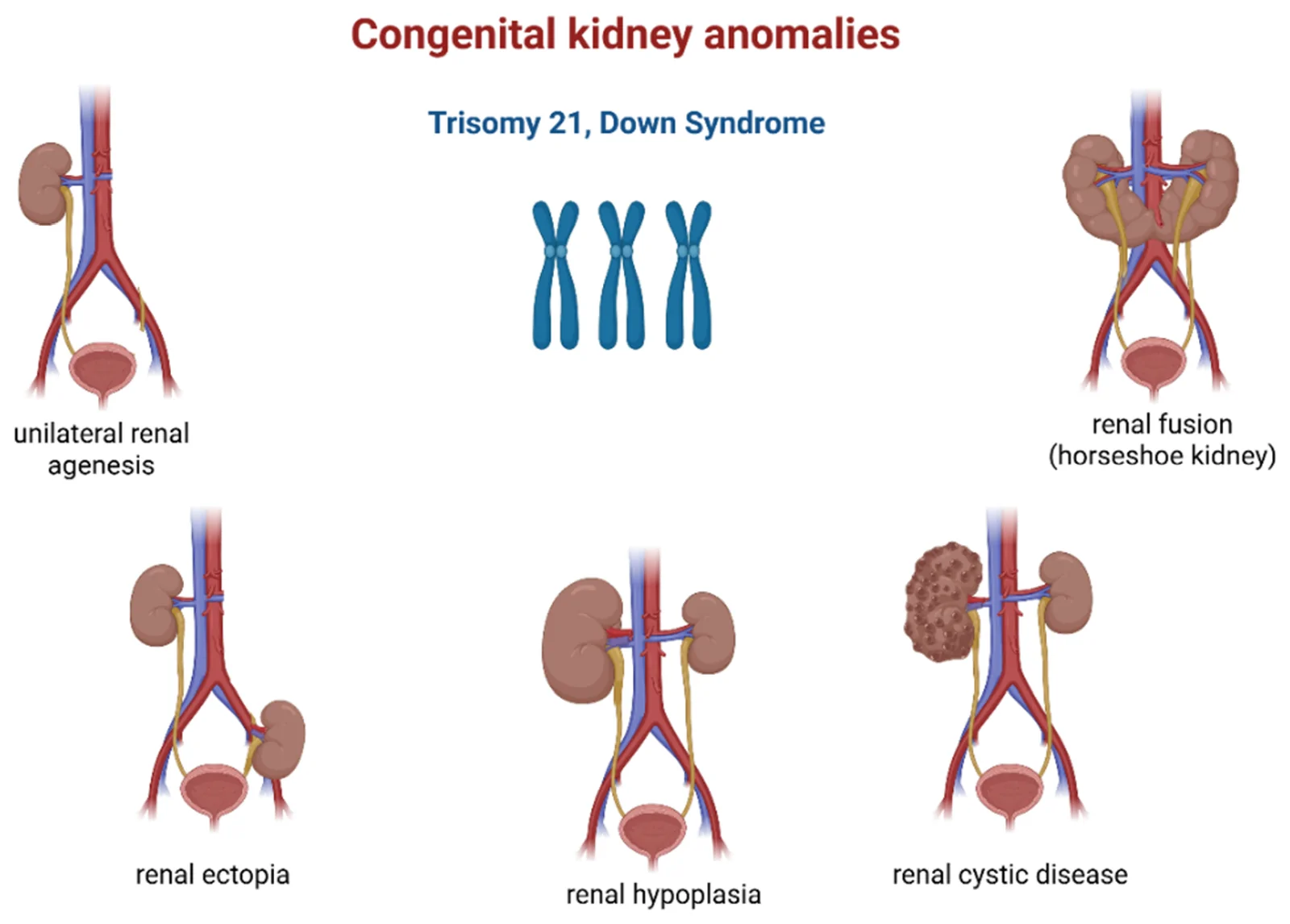
Spectrum of congenital kidney anomalies reported in Down syndrome.

Figure 2 was created by the authors using BioRender (BioRender.com) under an appropriate license (publication license provided by the authors).

## 3. Kidney Development and Nephrogenesis in Down Syndrome

### 3.1. Embryological Basis of Kidney Development and Nephrogenesis in Down Syndrome

Nephrogenesis is the complex embryological process of kidney formation and differentiation, involving reciprocal interactions between the ureteric bud and the metanephric mesenchyme and ultimately resulting in the development of the permanent kidney [9,10]. The urinary system primarily originates from the intermediate mesoderm, which forms a longitudinal elevation along the dorsal body wall known as the urogenital ridge. During embryonic development, three successive renal systems are formed in a craniocaudal sequence: the pronephros, mesonephros, and metanephros [11,12].

The pronephros is the earliest and most rudimentary nephric structure, appearing during the fourth week of gestation in the cervical region. It is nonfunctional in humans and rapidly regresses. The mesonephros subsequently develops caudally and functions transiently during early fetal life, contributing partially to the development of the male reproductive tract. The metanephros, which appears during the fifth week of gestation, gives rise to the permanent kidneys [9,12]. Development of the metanephros depends on tightly coordinated reciprocal signaling between two primordial structures: the ureteric bud, an outgrowth of the mesonephric duct, and the metanephric mesenchyme (also termed metanephric blastema). The ureteric bud gives rise to the collecting system, including the ureters, renal pelvis, calyces, and collecting ducts, while the metanephric mesenchyme differentiates into the nephrons, including Bowman’s capsule, proximal tubules, the loop of Henle, and distal tubules [10,13].

Multiple signaling pathways and transcription factors, including GDNF/RET, WT1, PAX2, EYA1, and WNT signaling, regulate ureteric bud branching morphogenesis and nephron induction, coordinating nephron number, renal architecture, and differentiation [14,15]. Nephrogenesis continues throughout fetal development and is largely completed between the 34th and 36th weeks of gestation, after which no significant new nephron formation occurs. Consequently, disturbances affecting embryonic renal development may result in congenital anomalies of the kidney and urinary tract (CAKUT) or reduced nephron endowment, potentially increasing susceptibility to renal dysfunction later in life [16,17].

Trisomy 21 is associated with widespread alterations in developmental regulation, raising the possibility that renal organogenesis may also be affected. However, the specific molecular mechanisms underlying abnormal nephrogenesis in Down syndrome remain insufficiently characterized, and direct experimental evidence from human fetal kidneys is limited. Although disturbances in nephrogenesis may contribute to congenital renal abnormalities, their potential relationship with subsequent renal dysfunction and acquired kidney diseases in individuals with Down syndrome remains to be established.

### 3.2. Morphological, Embryological and Molecular Evidence for Impaired Nephrogenesis in Down Syndrome

Trisomy 21 is characterized by disturbed developmental homeostasis, altered gene dosage effects, impaired cellular proliferation, and dysregulated differentiation, which contribute to abnormalities in multiple developing organs [3]. These mechanisms may also contribute to the alterations in fetal renal development observed in Down syndrome, although their specific involvement in nephrogenesis has not been established.

Since nephrogenesis is restricted to fetal life and nephron formation cannot be substantially restored after birth, developmental disturbances occurring during fetal kidney formation may have long-term consequences for renal reserve and susceptibility to chronic kidney disease [18].

Several perinatal characteristics associated with Down syndrome (DS) suggest a potential risk of reduced nephron endowment. Studies by Yao et al. [19] and Brock et al. [20] have shown that pregnancies affected by trisomy 21 are more frequently complicated by fetal growth restriction, preterm birth, small-for-gestational-age status, and other adverse perinatal outcomes than pregnancies in the general population. In addition, even term infants with DS often have lower birth weights than gestational age-matched controls. These perinatal factors may independently affect nephron development [18] and should therefore be considered potential confounders when interpreting renal morphological findings in DS. The available evidence does not allow their contribution to be reliably distinguished from developmental effects directly attributable to trisomy 21.

Morphological and histopathological studies provide additional evidence for altered fetal renal development in DS. The earliest evidence suggesting impaired fetal renal development came from Naeye et al. (1967) [21]. As summarized by Leskur et al. [5], an early autopsy study by Naeye reported reduced renal weight and an approximately 55% lower glomerular number in infants with Down syndrome compared with controls. However, these findings should be interpreted cautiously, as the diagnosis was based on phenotypic characteristics rather than cytogenetic confirmation, and the study involved a relatively small number of cases.

Histomorphometric studies subsequently demonstrated altered renal architecture during fetal development in Down syndrome. In a comparative fetal study, Desogus et al. found enlarged glomeruli, significantly widened Bowman’s spaces, and an expanded nephrogenic zone in DS kidneys compared with age-matched controls, along with a higher proportion of morphologically abnormal glomeruli [22]. In addition, DS fetal kidneys showed persistent immature nephronic structures and irregular glomerular morphology, suggesting delayed cortical maturation rather than normal progression of nephron differentiation.

Ariel extended these observations by showing persistent immature glomeruli in deeper cortical regions, whereas glomeruli in the deeper cortical maturation zone are expected to be more mature at the corresponding gestational age [23]. This abnormal spatial distribution is consistent with delayed maturation of the fetal renal cortex in Down syndrome. In a subsequent autopsy series comprising 124 individuals with Down syndrome of different ages, the same group reported glomerular microcysts as the most frequent renal histopathological abnormality, whereas other lesions, including focal tubular dilatation and simple cysts, were observed less consistently. However, the absence of a control group and the lack of clear diagnostic criteria for glomerular microcysts limit the interpretation of these findings [23].

Additional case-based observations further support abnormal nephrogenesis in DS. Wada et al. reported a patient with DS and oligonephronia whose renal biopsy demonstrated reduced glomerular density, mild tubular dilatation, and podocyte abnormalities consistent with immature renal development [24]. Renal tubular dysgenesis has also been reported in a hydropic fetus with trisomy 21. This rare and severe developmental disorder is characterized by a marked reduction or complete absence of proximal convoluted tubules despite relatively preserved glomerular and distal tubular development, leading to profound renal dysfunction and oligohydramnios-related complications [25].

Although direct studies investigating nephrogenesis-specific signaling pathways in human DS fetal kidneys remain limited, several developmental pathways essential for normal renal morphogenesis have been extensively characterized.

Nephron induction and ureteric branching are regulated through tightly coordinated interactions involving WT1, PAX2, GDNF/RET, EYA1, SIX2, and WNT signaling pathways, which collectively control nephron progenitor cell maintenance, epithelial differentiation, and branching morphogenesis [14]. Precise regulation of these pathways is essential for the establishment of an adequate nephron endowment, as disturbances in progenitor cell survival or differentiation may reduce nephron numbers at birth. Although researchers have extensively characterized key regulators of normal nephrogenesis, including WT1, PAX2, GDNF/RET, EYA1, SIX2, and WNT signaling pathways, their potential dysregulation in fetal kidneys with Down syndrome remains largely unexplored.

Trisomy 21 is associated with widespread gene dosage effects, altered transcriptional regulation, impaired cellular proliferation, and developmental dysregulation affecting multiple organ systems [26]. Particular attention has been directed toward DYRK1A, which has been implicated in embryonal tissue development, progenitor cell regulation, and organogenesis in experimental models [3]. Experimental evidence supports a role for DYRK1A in renal development. Blackburn et al. showed that reduced Dyrk1a expression disrupts embryonic nephron development in Xenopus laevis, while pathogenic loss-of-function variants in humans have been associated with congenital kidney and urinary tract anomalies [27]. However, these findings concern DYRK1A deficiency rather than the increased gene dosage associated with trisomy 21. Therefore, whether DYRK1A overexpression contributes to abnormal nephrogenesis in Down syndrome remains to be established. Furthermore, direct molecular evidence on how trisomy 21 regulates nephrogenesis-associated pathways in human fetal kidneys remains limited.

Lo et al. further complemented these observations by reporting reduced cortical development and abnormalities within the nephrogenic zone of fetal DS kidneys, consistent with altered renal maturation [28]. Together with the findings of other fetal histological studies, these observations suggest that renal development may be affected in trisomy 21. However, it remains uncertain whether these changes reflect DS-specific developmental mechanisms or the effects of associated conditions, including prematurity, intrauterine growth restriction, and congenital heart disease. Future studies combining fetal kidney morphometry with transcriptomic, epigenetic, and molecular analyses are needed to clarify the mechanisms underlying these abnormalities.

Taken together, the currently available evidence suggests that fetal renal development in DS may be characterized by altered glomerular morphology, delayed maturation, and abnormalities of nephrogenic structures. Although reduced nephron endowment is a recognized risk factor for subsequent renal dysfunction in the general population, its occurrence and long-term clinical consequences in individuals with DS remain insufficiently established. In particular, available studies have not demonstrated a direct longitudinal association between developmental renal abnormalities in DS and subsequent decline in kidney function, albuminuria, hypertension, or CKD. Therefore, the proposed relationship between impaired nephrogenesis and later renal disease in DS remains biologically plausible but requires prospective validation. Furthermore, much of the available morphological evidence derives from older autopsy studies, small fetal series, and isolated case reports, limiting the ability to distinguish DS-specific developmental alterations from the effects of associated perinatal conditions.

## 4. Congenital Anomalies of the Kidney and Urinary Tract (CAKUT) in Down Syndrome

CAKUT is a heterogeneous group of developmental abnormalities affecting the kidneys and urinary tract that can vary widely in location, severity, and the structures involved [5]. Currently, guidelines for DS still do not address renal and urinary tract anomalies and their role in the development of CKD. This is probably because CAKUT was previously considered uncommon in children with DS. However, several population-based studies report a higher prevalence of CAKUT among children with DS than in the general pediatric population, with relative estimates varying by study population, diagnostic criteria, and ascertainment methods [29,30,31]. For example, Kupferman et al. [30] reported a CAKUT prevalence of 3.2% in children with DS (OR 4.5, 95% CI 3.8–5.4), whereas Rankin et al. [32] reported 1.1% and Morris et al. [33] reported 2.3% for severe CAKUT phenotypes. However, these estimates are not directly comparable because of differences in study populations, the inclusion of fetal deaths and terminations of pregnancy, diagnostic definitions, and ascertainment methods. Moreover, studies do not consistently report confidence intervals and comparable effect estimates, limiting a more precise assessment of the association.

Significant congenital anomalies can often be clearly visualized by the 19th–20th weeks of gestation, necessitating careful prenatal and postnatal monitoring plans. Despite this, urinary tract infections (UTIs) are not more prevalent in children with DS than in the general population. Notably, certain CAKUT phenotypes may remain clinically silent, potentially delaying diagnosis and increasing the risk of renal complications [29].

### Kidney Anomalies in Individuals with DS

Congenital anomalies of the kidney and urinary tract (CAKUT) comprise structural abnormalities affecting kidney size, shape, position, or urinary tract development. This section focuses on congenital anatomical anomalies reported in individuals with Down syndrome, whereas developmental and histopathological abnormalities have been discussed in the previous section [5].

Renal agenesis is defined as the congenital absence of one or both kidneys. While bilateral agenesis is life-limiting, in unilateral renal agenesis the remaining kidney compensates through hyperfiltration from birth, which may contribute to early renal dysfunction, hypertension, and CKD [34]. The literature provides conflicting results regarding the prevalence of renal agenesis in DS compared with healthy controls. Three population-based studies reported prevalences of 0.03–0.04% but did not demonstrate a statistically significant increase compared with the general population [5]. However, methodological differences warrant cautious interpretation. Källén et al. combined renal agenesis and dysgenesis, preventing separate estimation of renal agenesis prevalence [35]. Torfs and Christianson considered renal agenesis separately but acknowledged that excluding stillbirths and terminations of pregnancy for fetal anomalies (TOPFAs) may have led to underestimation [36]. In contrast, Kupferman et al. reported a significantly higher prevalence of renal agenesis in children with DS than in controls (0.23% vs. 0.04%; OR 5.4, 95% CI 2.8–10.4) [30]. These differences highlight how study design, diagnostic classification, and population selection shape reported prevalence estimates.

Table 1 summarizes published studies reporting congenital anomalies, developmental abnormalities, and histopathological findings of the kidney in individuals with Down syndrome.

The definition of renal hypoplasia has undergone several changes. Today, the condition is defined by abnormally small kidneys, with a kidney volume below two standard deviations from the mean for age-matched healthy individuals [59] or a combined kidney volume less than half the expected volume for the patient’s age [5]. This inconsistency in diagnostic criteria calls for a careful approach to data from older studies. As reported by Leskur et al. [5], an early autopsy study by Naeye (1967) [21] reported a 31.1% reduction in renal mass in infants classified as having DS compared with controls matched for gestational age and body weight. More recently, Postolache et al. demonstrated significantly smaller kidney length and volume in children with DS compared with age- and sex-matched controls [58]. Importantly, these differences remained statistically significant after adjustment for body surface area, indicating that reduced renal dimensions cannot be explained solely by differences in overall body size.

Furthermore, measured kidney length in children with DS was significantly lower than the expected length calculated using age, height, and weight. Nevertheless, the authors acknowledged that the reference equation had not been validated specifically in children with DS, and smaller renal dimensions do not necessarily establish pathological renal hypoplasia. Given the distinct growth patterns and body composition of children with DS, along with reported differences in renal function parameters, these findings should be interpreted in the context of DS-specific anthropometric and clinical characteristics [60,61,62,63].

Although an earlier population-based study did not identify a significantly increased prevalence of renal agenesis/hypoplasia in DS [38], more recent investigations have reported reduced renal dimensions in affected children. Shimozono et al. also reported reduced renal dimensions in children with DS undergoing cardiac surgery [64]. In a cross-sectional study of 54 children with DS, Padoan et al. reported renal hypoplasia in about 26% of participants (14/54), defined as kidney length below the third percentile of age-matched reference values [59]. The authors also observed reduced eGFR, with approximately 26% of children having an eGFR below 90 mL/min/1.73 m^2^ and 9.3% below 75 mL/min/1.73 m^2^. Among adolescents, 5 of 9 (55.5%) had an eGFR below 90 mL/min/1.73 m^2^. However, the authors found no statistically significant association between kidney size, BMI, and eGFR.

Furthermore, kidney dimensions were evaluated using age-based reference values, without reported adjustment for height or body surface area. These findings support the importance of assessing both renal size and function in children with DS, while highlighting the need for larger studies incorporating anthropometric adjustments and longitudinal follow-up [65]. Importantly, reduced renal dimensions alone do not necessarily indicate pathological renal hypoplasia, as they may partly reflect the characteristic growth patterns of children with DS. Although Postolache et al. [58] suggest that smaller kidneys cannot be explained solely by differences in body size, the available studies do not reliably distinguish true developmental renal hypoplasia from proportionally smaller kidneys without clinically significant structural or functional abnormalities.

Renal hypoplasia and dysplasia are recognized causes of chronic kidney disease in the general pediatric population, accounting for 14.2% of primary renal lesions among children receiving dialysis. Given the reported frequency of reduced renal dimensions in children with DS [59], these findings raise concerns about potential long-term renal vulnerability. However, current evidence does not establish whether smaller kidneys in DS independently predict progressive renal dysfunction or CKD. Longitudinal studies are needed to determine whether developmental renal abnormalities in DS are associated with subsequent deterioration of renal function, albuminuria, hypertension, or CKD. Therefore, careful assessment of renal size and function during follow-up of children with DS may help identify clinically significant renal abnormalities.

Renal dysplasia is a developmental abnormality characterized by abnormal renal differentiation and disorganized renal architecture [66]. It may result from intrinsic disturbances in renal differentiation or develop with functional or structural obstruction of the lower urinary tract [67]. Differences in diagnostic criteria and potential overlap among renal dysplasia, renal hypoplasia, and cystic renal disease may complicate cross-study comparisons and contribute to uncertainty in reported prevalence estimates. Renal dysplasia was reported by Morris et al. in a large European population-based registry study, although the diagnostic criteria used to distinguish dysplasia from other renal malformations were not specified. The study included 7044 live births and fetal deaths with DS registered in 28 European population-based congenital anomaly registries covering approximately seven million births between 2000 and 2010. Among these DS cases, 43.6% had a cardiac anomaly and 15.0% had a non-cardiac anomaly [33]. Renal dysplasia was also described in a case report of a female child in whom postnatal abdominal ultrasonography revealed a megabladder and dysplastic kidneys [50].

Renal cystic abnormalities have been reported in individuals with DS, although their prevalence and clinical significance remain uncertain. Ariel et al. (1991) [23] examined 124 autopsy cases of Down syndrome. Among the cases with available renal findings, glomerular microcysts were identified in 23 of 97 cases and simple cysts in 7 of 97 cases [23]. Stanciu et al. reported a case of a 13-week-gestation fetus with mild hydronephrosis of the right kidney, while the left kidney was not visualized on ultrasound. Following genetic confirmation of DS and termination of pregnancy, post-mortem MRI revealed several scattered simple cystic structures within the renal cortex of the right kidney and hypoplasia of the left kidney [44]. Another case report described a 3.5-month-old infant with DS and polycystic kidney disease who developed early end-stage renal failure. The author acknowledged that these conditions could coexist coincidentally and that any potential association requires further investigation [68].

With the advent of routine prenatal imaging, the detection of renal anomalies has increased; however, the underlying etiologies and clinical significance of some findings remain unclear. This is particularly relevant to the prenatal diagnosis of cystic renal abnormalities. The terms “cystic kidney disease” and “renal cystic dysplasia” encompass a heterogeneous group of renal conditions. Although renal cystic dysplasia in infants shares several features with multicystic dysplastic kidney (MCDK), distinguishing MCDK from other causes of renal cysts is important for accurate diagnosis, appropriate counseling, and clinical management [69].

Other less frequently reported renal anomalies in DS include ectopic and horseshoe kidneys. Ectopic kidneys, which fail to reach their normal anatomical position, may be associated with hydronephrosis and impaired renal function [70]. Early studies described horseshoe kidney, a congenital renal fusion anomaly, as part of the DS phenotype, but subsequent research has given it relatively limited attention [71]. Horseshoe kidney is frequently associated with additional urinary tract abnormalities and impaired urinary drainage. Its reported incidence in the general population ranges from approximately 0.15% to 0.25% [72,73], while the population-based study by Torfs and Christianson reported a significant association between DS and horseshoe kidney (risk ratio 11.7, 0.001 < *p* < 0.05), with a prevalence of 0.07% [36].

## 5. Acquired Glomerulopathies and Pathohistological Renal Lesions in Patients with DS

Acquired glomerulopathies have been reported in individuals with Down syndrome. However, available data, as with CAKUT, come primarily from case reports or case series. It is important to highlight all documented glomerulopathies in this population; however, without larger studies and population-based surveys, it is not possible to determine whether their incidence differs from that in the general population [7,28].

The terminology used to describe non-congenital renal involvement in Down syndrome is not entirely consistent across the literature. Terms such as acquired glomerulopathies, acquired renal disease, secondary renal lesions, and secondary kidney damage are sometimes used interchangeably despite referring to different pathological processes. In a strict nephrological context, acquired glomerulopathies refer to glomerular disorders that develop after birth and are not directly related to congenital malformations. However, some authors emphasize that congenital anomalies of the kidney and urinary tract (CAKUT) may predispose individuals with Down syndrome to subsequent acquired renal injury through urinary tract obstruction, recurrent infections, hypertension, or progressive structural damage [5].

A different conceptual approach was proposed by Ranchin et al. [7], who distinguished primary renal disease from secondary renal injury. In their classification, secondary lesions comprise renal dysfunction associated with conditions that increase the risk of chronic kidney disease and occur more frequently in individuals with Down syndrome, including prematurity, low birth weight, small-for-gestational-age status, congenital heart disease, and other comorbidities. Consequently, distinguishing acquired from secondary renal lesions is not always straightforward, and available studies often include findings from both categories. For this review, acquired renal disease refers to renal disorders that develop after birth, whereas secondary renal injury refers to kidney damage occurring as a consequence of associated congenital anomalies or systemic comorbidities.

### 5.1. Comorbidities and Secondary Renal Injury in Down Syndrome

Other conditions more common in DS may increase the risk of chronic kidney disease. These include prematurity, low birth weight, small-for-gestational-age status, and congenital heart disease [7].

Congenital heart defects are among the most common comorbidities in individuals with DS, affecting over 40% of patients, with female individuals more likely to have cardiac anomalies [3,33]. In addition to the direct cardiovascular consequences, these conditions could contribute to renal vulnerability through altered systemic and renal hemodynamics, impaired renal perfusion, and complications associated with cardiac surgery.

Shimozono et al. [64] conducted a retrospective study of infants undergoing cardiac surgery for congenital heart disease. They reported smaller kidney size and less favorable preoperative renal function parameters in infants with DS compared with infants without DS. However, the incidence and severity of cardiac surgery-associated acute kidney injury did not differ significantly between the groups. These findings suggest that pre-existing renal differences in infants with DS do not necessarily translate into an increased risk of perioperative acute kidney injury.

Prematurity, low birth weight, and small-for-gestational-age status may further contribute to renal vulnerability through reduced nephron endowment and increased susceptibility to subsequent kidney injury [7,16]. Other systemic conditions associated with DS, including type 1 diabetes mellitus and hematological malignancies, may also affect renal health through disease-related complications or treatment-associated nephrotoxicity [3]. Nevertheless, their specific contribution to chronic kidney disease in individuals with DS remains insufficiently investigated.

In a large retrospective cohort study, Huang et al. [6] reported a significantly higher risk of chronic kidney disease (HR 2.7, 95% CI 2.1–3.5), hypertension (HR 1.6, 95% CI 1.4–1.8), and ischemic heart disease (HR 3.7, 95% CI 3.0–4.6) in individuals with DS compared with matched controls over 10 years. Although these findings indicate an increased burden of non-congenital renal and cardiovascular conditions, they do not establish whether renal impairment results directly from DS-associated developmental abnormalities or is mediated by congenital anomalies and systemic comorbidities. Therefore, clinicians should consider these factors in assessing renal dysfunction and as potential confounders in studies investigating kidney outcomes in DS.

Recent population-based evidence further supports the clinical relevance of renal complications in Down syndrome. In a nationwide Danish cohort study, Weldingh et al. (2026) analyzed 2815 individuals with Down syndrome with available serum creatinine measurements and compared them with age- and sex-matched individuals from the general population [74]. The cumulative risk of acute kidney injury by age 40 years was 32.8% in individuals with Down syndrome compared with 5.3% in controls, while the corresponding risk of chronic kidney disease was 3.9% versus 0.4%. Importantly, the increased risks persisted among individuals without congenital heart disease, suggesting that renal vulnerability in Down syndrome cannot be attributed solely to associated cardiac abnormalities. Nevertheless, because both outcomes were defined using creatinine-based criteria, these findings should be interpreted in light of the known limitations of creatinine-based renal assessment in this population.

### 5.2. Acquired Glomerulopathies Reported in Persons with Down Syndrome

Early autopsy studies occasionally described renal histopathological abnormalities in individuals with Down syndrome; however, most were primarily focused on congenital malformations and were limited by the absence of control groups, heterogeneous age distribution, and non-standardized diagnostic criteria [8,54].

One of the earliest reports of acquired glomerulopathy in individuals with DS was published by Gupta et al. in 1991 [39]. The study described four cases of membranoproliferative glomerulonephritis (MPGN) in adult males aged 20–30 years [39]. Subsequently, only a few additional cases were reported [75], with fewer than 10 cases reported in total [5].

Another early study was conducted by Takemura et al., who reported immunotactoid glomerulopathy (fibrillary glomerulonephritis) in a 9-year-old girl with DS, who had presented with proteinuria and microscopic haematuria at the age of 6 years. Renal biopsy revealed characteristic histopathological findings, including glomerular basement membrane thickening, diffuse mesangial expansion, and site-specific deposition of IgG antibodies and complement components [76].

Until 1998, focal segmental glomerulosclerosis (FSGS) had not been described in DS. Unlike MPGN, whose pathogenesis is predominantly immune-complex mediated, FSGS raised the question of whether glomerular injury in DS might also involve podocyte damage. Lo et al. and Baqi et al. independently reported the first two cases in 1998 [28,77]. Lo et al. reported two cases in males aged 17 and 19 years who presented with renal failure, without a previous history of renal illness. It could not be determined whether FSGS was primary or secondary in these cases, although the collapsing variant is generally considered primary.

These early studies indicated that acquired glomerular disease might be heterogeneous rather than disease-specific. Major contribution to understanding this spectrum was provided by the biopsy series conducted at the Mayo Clinic and reported by Said et al., who thoroughly evaluated acquired glomerular lesions in patients with DS [75]. One major contribution of this study was that the authors not only identified DS-associated nephropathies but also demonstrated a heterogeneous spectrum of acquired glomerular diseases, indicating that no single histopathological pattern predominates in these patients.

IgA nephropathy was the most frequently identified acquired glomerulopathy on renal biopsy (29%) in a retrospective study of individuals with DS [75]. Reported morphological lesions included focal cellular crescents in one case, mild focal dilatation of the urinary space in four cases, and, in a patient with diabetes, underlying diabetic glomerulosclerosis manifesting as diffuse mesangial sclerosis and thickening of the glomerular basement membrane.

Pauci-immune crescentic glomerulonephritis has been reported in a total of four cases to date [5,75] with severe clinical presentations, including rapidly progressive renal failure and biopsy findings consistent with ANCA-associated vasculitis [78].

Membranous glomerulonephritis has been reported in only two male individuals with DS, aged 27 and 14 years, with diagnosis confirmed by typical morphological and immunofluorescence findings [28,75]. An equal number of cases of acute postinfectious glomerulonephritis have been reported, involving patients aged 6 and 41 years. Lupus nephritis has been described in only one patient with DS to date, who had previously been diagnosed with systemic lupus erythematosus. Characteristic findings included mesangial hypercellularity, focal cellular crescents, and segmental mesangial and subendothelial electron-dense deposits [75].

Renal amyloidosis was reported by Ozkaya et al. [79] in 2005 in a patient with DS and pulmonary tuberculosis who presented clinically with nephrotic syndrome. The diagnosis was confirmed by renal biopsy, which revealed diffuse Congo red-positive mesangial amyloid deposits [79].

Other rare types of acquired glomerulopathies include antineutrophil cytoplasmic antibody (ANCA)-associated glomerulonephritis, reported in three individuals with DS [80,81,82], and IgG glomerulonephritis, reported only once in a 17-year-old girl with DS in the absence of an identifiable autoimmune disorder or any other etiological factors known to cause secondary IgG-associated glomerulonephritis [83].

These observations raise several important issues that warrant further consideration and research. Although uncommon, acquired renal disease has been documented in individuals with DS. With increasing life expectancy, a growing number of individuals with DS may be at risk of CKD and end-stage renal disease, highlighting the importance of assessing renal health in this population.

A heterogeneous spectrum of acquired glomerular diseases has been documented in individuals with Down syndrome. Based on the evidence to date, we cannot establish with certainty an increased incidence or specific predisposition to any particular glomerulopathy in individuals with DS. Most published observations are based on case reports and small case series involving no more than 10 patients. Although one study described the clinicopathological characteristics of 17 patients with DS who underwent renal biopsy, the relative frequency of histopathological diagnoses among biopsied patients cannot be interpreted as their prevalence in the overall Down syndrome population. The most common glomerular disease identified on biopsy was IgA nephropathy among young adults who underwent kidney biopsy for proteinuria, hematuria, and renal insufficiency. This finding reflects the frequency of this particular glomerular disease among the renal biopsies analyzed. However, IgA nephropathy is also one of the most common renal conditions in the general population, and these data should not be used to infer its prevalence in the DS population. In addition to IgA nephropathy, numerous immune-complex, podocytopathic, pauci-immune, and other rare glomerular disorders have been described. This diversity may partly reflect the increased susceptibility of individuals with Down syndrome to immune dysregulation [84] and autoimmune disease. Research indicates that a coexisting autoimmune condition, particularly hypothyroidism, is frequently present in these patients, but convincing epidemiological evidence demonstrating an increased overall incidence of acquired glomerulopathies remains lacking [75]. Furthermore, despite the diversity of glomerular lesions, primary tubulointerstitial and vascular diseases have been reported only exceptionally, suggesting that glomerular involvement remains the predominant pattern of acquired renal pathology in Down syndrome [75,85].

This summary aims to document the range and heterogeneity of acquired glomerular diseases reported in individuals with Down syndrome, not to define a disease-specific pattern. It is also important to emphasize that renal abnormalities in this population should not automatically be attributed to congenital anomalies or developmental renal impairment. Population-based studies with adequate comparison groups are needed to determine whether any of these glomerulopathies occur more frequently in individuals with DS. Further studies are needed to elucidate immunological abnormalities in patients with DS and glomerular disease.

## 6. Assessment of Renal Function and Emerging Biomarkers in Down Syndrome

Assessment of renal function in individuals with Down syndrome (DS) presents several challenges because conventional biomarkers may not accurately reflect true kidney function in this population. Serum creatinine (sCr) and estimated glomerular filtration rate (eGFR) remain the standard clinical tools for renal assessment [86,87]. However, their interpretation in DS is complicated by reduced muscle mass, altered body composition, shorter stature, smaller kidney size, and distinct growth patterns compared with the general population [58,60,61,62,63,75,85,87,88,89]. Several authors have raised concerns about applying commonly used creatinine-based pediatric eGFR equations in individuals with Down syndrome because this population’s anthropometric and body composition characteristics may affect the accuracy of creatinine-based renal function estimates [58,62,90,91]. However, direct validation of commonly used eGFR equations against measured GFR in individuals with Down syndrome remains scarce. Consequently, it has not yet been conclusively established whether conventional creatinine-based equations systematically overestimate renal function in this population.

Recent studies have further highlighted the challenges of interpreting serum creatinine concentrations in children with Down syndrome. In a retrospective study of 194 children, Farrell et al. (2026) [92] reported serum creatinine concentrations above standard pediatric reference limits in 26.8% of participants, while 34% of those with available height measurements had an eGFR below 90 mL/min/1.73 m^2^ [92]. In a separate cohort of 523 children and adolescents with Down syndrome, Gür et al. (2026) [93] established age- and sex-specific serum creatinine centile curves based on 2172 measurements, demonstrating age-related changes and increasing differences between sexes during adolescence [93]. Together, these findings support the need to interpret conventional creatinine reference ranges carefully in this population. However, syndrome-specific creatinine centiles do not establish the accuracy of eGFR equations, which still require validation against measured GFR.

It has also been proposed that reduced nephron endowment, smaller renal volume, and developmental renal abnormalities described in DS may contribute to diminished renal functional reserve throughout life. However, researchers have not yet directly established this relationship in individuals with DS [58,90]. Furthermore, increased oxidative stress and accelerated biological aging, both recognized features of DS, may predispose affected individuals to earlier renal decline [94,95]. Despite these concerns, routine nephrological screening and standardized renal monitoring protocols remain insufficiently defined in currently available healthcare guidelines for individuals with DS [4,7,85]. Currently, evidence is insufficient to support a specific, evidence-based screening strategy for the entire Down syndrome population. Consequently, the optimal screening approach remains unclear. Prospective validation is needed to determine whether renal ultrasound, blood pressure monitoring, albuminuria testing, serum creatinine, cystatin C, or combinations of these approaches should be routinely applied to all individuals with DS or reserved for selected high-risk groups.

These limitations of conventional renal function assessment have stimulated interest in biomarkers capable of detecting renal injury at earlier stages, particularly in populations with altered body composition such as individuals with Down syndrome.

Interest in novel biomarkers for early renal injury detection in DS remains limited but is gradually increasing. Among emerging biomarkers, neutrophil gelatinase-associated lipocalin (NGAL) and kidney injury molecule-1 (KIM-1) have attracted attention because they may detect renal injury earlier than conventional markers. NGAL is considered a sensitive indicator of tubular injury and systemic inflammation, while KIM-1 reflects proximal tubular epithelial damage [86,96]. However, evidence regarding their utility specifically in DS is scarce. Dogliotti et al. reported mildly elevated NGAL levels in children with DS, particularly in older individuals, suggesting either chronic low-grade inflammation or increased susceptibility to early renal injury [97].

In contrast, studies specifically evaluating KIM-1 in DS populations are currently lacking. Therefore, NGAL and KIM-1 should currently be regarded as promising investigational biomarkers rather than validated tools for routine clinical assessment in individuals with Down syndrome. While NGAL may provide additional information regarding early renal injury, its interpretation may be confounded by the chronic low-grade inflammatory state frequently observed in DS. Similarly, the potential utility of KIM-1 remains largely hypothetical without DS-specific studies. Future prospective studies are needed to determine whether these biomarkers provide additional diagnostic or prognostic value beyond conventional methods of renal assessment in this population.

Cystatin C has been widely investigated as an alternative filtration marker because, unlike serum creatinine, it is less influenced by muscle mass and body composition. This characteristic may be particularly advantageous in individuals with Down syndrome, who often exhibit reduced lean body mass and altered body composition. However, thyroid dysfunction, a common comorbidity in Down syndrome, may also affect cystatin C concentrations, potentially influencing interpretation independently of renal function [98]. Consequently, combined creatinine-cystatin C equations may offer theoretical advantages by reducing the limitations associated with either biomarker alone. Nevertheless, studies specifically validating cystatin C-based or combined equations in individuals with Down syndrome remain limited.

Consequently, future prospective longitudinal studies that integrate conventional renal function assessment with emerging biomarkers such as NGAL and KIM-1, along with renal morphometry and developmental imaging, may substantially improve early detection of renal vulnerability in individuals with Down syndrome.

Clinically, renal assessment in individuals with Down syndrome currently relies on established approaches, including clinical evaluation, serum creatinine measurement, estimated glomerular filtration rate (eGFR), urinalysis, and renal ultrasonography when clinically indicated. However, creatinine-based eGFR estimates should be interpreted cautiously because differences in muscle mass and body composition may affect their accuracy. Cystatin C may provide complementary information, although cystatin C-based equations have not been adequately validated in this population. In contrast, urinary biomarkers such as NGAL and KIM-1 remain investigational in Down syndrome and cannot currently be recommended for routine renal screening. Although recent studies have provided Down syndrome-specific serum creatinine reference data, further research is needed to validate methods for assessing glomerular filtration and to establish evidence-based strategies for the early detection and monitoring of renal dysfunction in this population.

## 7. Synthesis and Strength of Available Evidence

This review’s primary contribution is integrating existing knowledge and data on developmental processes, morphological characteristics, and current clinical evidence of renal involvement in Down syndrome. It is crucial to analyze and highlight both the strengths and limitations of the available data and their sources.

Data on the prevalence of congenital renal anomalies come from population-based studies, whereas information on potential impairment in nephrogenesis comes primarily from smaller histological and autopsy-based studies.

Data concerning acquired glomerulopathies are limited to individual case reports and selected biopsy series, which precludes drawing definitive conclusions about their prevalence within the population of individuals with Down syndrome. Furthermore, evidence on the utility of emerging renal biomarkers is extremely scarce, and strategies for Down syndrome-specific screening are either nonexistent or, at best, inadequate. This distinction between established findings and associations that remain plausible hypotheses rather than established causal relationships underscores the need for prospective, longitudinal investigations.

## 8. Conclusions

As indicated by current evidence, renal involvement in Down syndrome is not restricted to isolated congenital anomalies but rather reflects complex interactions between impaired renal development, congenital structural abnormalities, acquired renal disease, and systemic comorbidities.

Findings from small fetal histological and autopsy studies suggest delayed renal maturation and possible alterations in nephron endowment in individuals with Down syndrome. However, it remains unclear whether these developmental abnormalities contribute to subsequent CKD, as no direct causal relationship has been established.

Future research should integrate fetal kidney morphometry, transcriptomic and epigenetic profiling, advanced imaging, and longitudinal assessment of renal function to clarify the developmental origins of renal vulnerability in Down syndrome and to establish evidence-based strategies for early diagnosis and long-term nephrological surveillance.

## Figures and Tables

**Table 1:** Reported congenital, developmental and histopathological renal abnormalities in individuals with Down syndrome.

Authors	Year	Study Design	Population/Sample	Main Renal Findings	Control Group
Berg et al. [8]	1960	Autopsy study	141 autopsies	RA (2), RH (2), RF (1)	No
Insunza et al. [37]	1993	Case report	1 patient	RA (1)	No
Källén et al. [35]	1996	Population-based infant study	5581 infants	RA/RD (2)	Not specified
Torfs and Christianson [36]	1998	Population-based infant study	2894 infants	RA (1), RF (2)	No
Clevens et al. [38]	2007	Population study	43,463 infants	RA/RH (14)	No
Kupferman et al. [30]	2009	Infant cohort study	3832 infants	RA (9), RE (1), RCD-RDwCy (3), HyN (69)	No
Kupferman et al. [39] *	1994	Case reports	2 patients	HyN (2)	No
Morris et al. [33]	2014	Population study	14,109 live births/fetal deaths	RA (2), RD (10), HyN (45)	No
Ariel et al. [23]	1991	Autopsy histopathological study	124 autopsies	RH (18), RCD-GCyD (23), RCD-SRCy (7), RCD-RDwCy (4), HyN (3)	No
Gupta et al. [40]	1991	Case report	2 patients	RH (2)	No
Lo et al. [28]	1998	Autopsy histopathological study	45 autopsies	RH (1), RCD-GCyD (28)	Yes
Malaga et al. [41]	2005	Cross-sectional study	69 patients (12 months–24 years)	RH (1), RE (1), PyE (1)	No
Ebert et al. [42]	2008	Clinical imaging study	24 patients	RH (2), HyN (7)	No
Kute et al. [43]	2013	Case report	1 patient	RH (1)	No
Jain et al. [25]	2014	Clinical cohort	40 patients	HyN (8)	Not specified
Staicu et al. [44]	2015	Case report	1 patient	RH (1), RCD-SRCy (1), HyN (1)	No
Boronat et al. [45] *	1982	Case report	1 patient	RE (1)	No
Stein et al. [46]	1999	Case report	1 patient	RE (1)	No
Navarro et al. [47]	2005	Case report	1 patient	RE (1)	No
Al-Manjomi et al. [48]	2005	Case report	1 patient	RE (1)	No
Kosmadakis et al. [49]	2013	Case report	1 patient	RE (1)	No
Safdar et al. [50]	2018	Clinical cohort	241 patients	RE (5), HyN (18)	No
Stoll et al. [51] *	1990	Population study	139 cases	RF (1)	Not specified
Naeye et al. [21] *	1967	Autopsy study	21 autopsies	RF (1), HyN (1)	Yes
Anriquez et al. [52]	2023	Case report	1 patient	RD (1)	No
Webb et al. [53] *	1993	Case report	1 patient	RCD-RDwCy (1)	No
Egli and Stalder [54] *	1973	Autopsy study	103 autopsies	HyN (1)	No
Narashiman et al. [55]	2005	Clinical series	6 patients	HyN (4)	No
Hicks et al. [56]	2007	Case series	7 patients	HyN (7)	No
Weijerman et al. [57]	2008	Infant cohort	176 infants	HyN (2)	No
Rankin et al. [32]	2012	Population study	1115 stillbirths/miscarriages/TOPFAs	HyN (12)	Not specified
Postolache et al. [58]	2022	Pediatric cohort	49 children	HyN (2), PyE (3)	No
Padoan et al. [59]	2025	Pediatric cohort	54 children (66–123 months)	RH (14)	No

Abbreviations: RA—renal agenesis; RH—renal hypoplasia; RF—reduced nephron number/fetal renal growth impairment; RD—renal dysplasia; RE—renal ectopia; RCD-GCyD—renal cystic disease with glomerular cystic dysplasia; RCD-SRCy—renal cystic disease with subcapsular renal cysts; RCD-RDwCy—renal dysplasia with cysts; HyN—hydronephrosis; PyE—pyeloectasia; TOPFA—termination of pregnancy for fetal anomaly. **Notes:** Some studies reported multiple coexisting renal anomalies in the same patient. Exact numerical data were not always available because several original studies reported overlapping findings or incompletely specified denominators. ‘Not specified’ indicates that the presence of a control group or precise numerical information was not clearly reported in the original publication. * Data from the original study were obtained from the review by Leskur et al. (2025) [5], as the original publication was not directly accessed.

## Data Availability

No new data were created or analyzed in this study. Data sharing is not applicable to this article.
